# Rattlesnake (*Crotalus* spp.) distribution and diversity in Zacatecas, Mexico

**DOI:** 10.3897/zookeys.1005.56964

**Published:** 2020-12-18

**Authors:** Jesús Lenin Lara-Galván, Juan Felipe Martínez-Montoya, José Jesús Sigala-Rodríguez, Citlalli Edith Esparza-Estrada, Octavio César Rosas-Rosas, Lucía Ávila-Herrera, A. Márcia Barbosa

**Affiliations:** 1 Colegio de Postgraduados, Campus San Luis Potosí, Posgrado en Innovación en Manejo de Recursos Naturales. Iturbide 73, Salinas de Hidalgo, San Luis Potosí, CP. 78622, México Colegio de Postgraduados Salinas de Hidalgo Mexico; 2 Colección Zoológica. Universidad Autónoma de Aguascalientes. Aguascalientes, Ags. CP. 20131, México Universidad Autónoma de Aguascalientes Aguascalientes Mexico; 3 Instituto de Ecología, A.C., Red de Biología Evolutiva, Laboratorio de Macroecología Evolutiva. Carretera Antigua a Coatepec 351, El Haya, Xalapa, CP. 91070, Veracruz, México Instituto de Ecología, A.C. Xalapa Mexico; 4 Presidencia Municipal de Valparaíso. Constitución Sn, Capulín de la Sierra, CP. 99200. Valparaíso, Zacatecas, México Presidencia Municipal de Valparaíso Valparaíso Mexico; 5 CIBIO/InBIO, Universidade de Évora. 7004-516. Évora. Portugal Universidade de Évora Évora Portugal; 6 CICGE – Centro de Investigação em Ciências Geo-Espaciais, Universidade do Porto, Portugal Universidade do Porto Porto Portugal

**Keywords:** Central northern Mexico, conservation, Geographic Information System (GIS), herpetofauna, Species Distribution Models (SDM)

## Abstract

Mexico is home to a large number of reptile species and has one of the greatest diversities of venomous snakes, among which the rattlesnakes pertaining to the *Crotalus* genus stand out. Out of more than 40 species in the country, nine are found in Zacatecas: *C. aquilus*, *C. atrox*, *C. basiliscus*, *C. lepidus*, *C. molossus*, *C. polystictus*, *C. pricei*, *C. scutulatus* and *C. willardi*. Although these reptiles are important, due to their relevance in terms of ecology, cultural use and public health, their conservation is impacted by multiple factors, such as habitat fragmentation and indiscriminate killing. Thus, most species within this genus are found in some type of risk category at both the national and international level. The purpose of this study was to determine the potential distribution and diversity of rattlesnakes at the municipal level in the understudied state of Zacatecas. To do this, we analyzed and described the global distribution of nine rattlesnake species by building species distribution models, which determined their potential distribution based on a set of ecological variables and presence records. The resulting models were used to assess the diversity of rattlesnake species potentially present in each municipality within the state. Thirty-nine (67.24 %) out of fifty-eight municipalities registered at least one rattlesnake species. Fresnillo, Sombrerete and Valparaíso were some of the municipalities showing greatest diversity. Moreover, *C. atrox*, *C. lepidus*, *C. molossus* and *C. scutulatus* were the most widely found species in the state. On the other hand, *C. basiliscus*, *C. polystictus*, *C. pricei* and *C. willardi* were rarely spotted and so, information on their distribution patterns within Zacatecas is limited. Finally, the areas having the largest potential for the distribution of these species were defined. These findings should make field work much more time- and cost-effective, facilitating the collection of in situ data that are useful for management and conservation plans of these species in Zacatecas.

## Introduction

**Figure 1.**
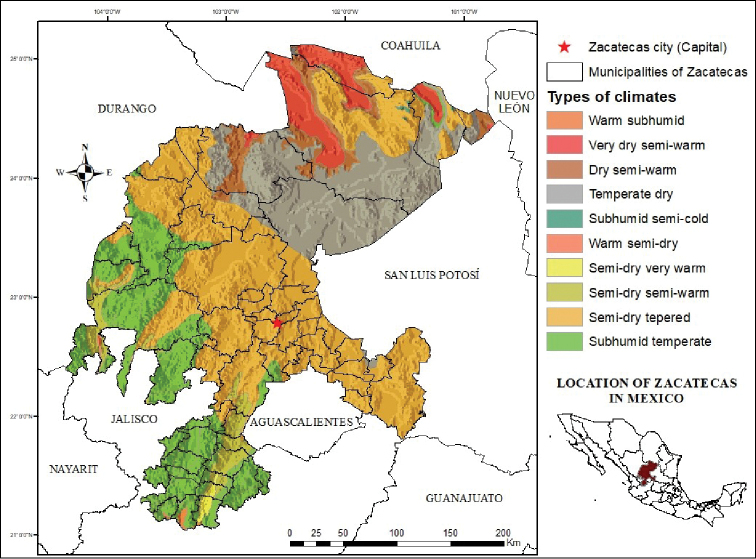
Location of the state of Zacatecas and their climate types (modified from INEGI 2008).

Mexico is home to a great diversity of reptile species, including a large number of endemics, with 864 different species having been reported by 2014 (Flores-Villela and García-Vázquez 2014). The states of Oaxaca (262), Chiapas (220) and Veracruz (200) have the greatest number of species (Flores-Villela and García-Vázquez 2014), while Zacatecas has only 108 according to the most recent information gathering (Sigala-Rodríguez et al. 2020). Pitvipers stand out among all these reptiles (Jadin et al. 2011) and, particularly in this family, rattlesnakes belonging to the *Crotalus* genus are noteworthy. As of 2018, this genus is comprised of 47 species, with 42 of them found in Mexico, among which 27 are endemic (Uetz 2018). Recently, there have been several studies on the herpetofauna in the central and northern regions of Mexico, in which Zacatecas is located. This state is one of the largest and shares its borders with eight other states (Figure 1). Despite its size, the data available concerning its herpetofauna is very scarce, thereby constituting an information gap about the species distributed in the region; Flores-Villela and García-Vázquez (2014) and Lemos-Espinal et al. (2018) underlined the lack of studies and species lists of herpetofauna in Zacatecas. By contrast, neighboring states have complete works on their amphibians and reptiles, such as Aguascalientes: Vázquez-Díaz and Quintero-Díaz 2005; Coahuila: Lemos-Espinal and Smith 2016a; Durango: Valdez Lares et al. 2013, Lemos-Espinal et al. 2018a, Lemos-Espinal et al. 2019; Guanajuato: Elizalde-Arellano et al. 2014; Jalisco: Chávez-Avila et al. 2015; Nayarit: Luja et al. 2014, Woolrich-Pina et al. 2016; Nuevo León: Lazcano-Villarreal 1997; Lazcano-Villarreal et al. 2010; Lemos-Espinal et al. 2016b; Lemos-Espinal et al. 2018b; and San Luis Potosí: Lemos-Espinal et al. 2018c.

Another problem that Zacatecas faces is the lack of publications on regional or municipal studies with venomous snakes and herpetofauna in general. There are only specific notes recorded on the state rattlesnake species of the *Crotalus* genus such as *C. aquilus* (Carbajal-Márquez et al. 2015a), *C. basiliscus* (Ahumada-Carrillo et al. 2011; Carbajal-Márquez et al. 2015b); *C. polystictus* (Campbell and Lamar 2004; Ahumada-Carrillo 2010), *C. atrox*, *C. lepidus*, *C. molossus* and *C. scutulatus* (Campbell and Lamar 2004), *C. willardi* (Klauber 1949). Moreover, the presence of *C. pricei* in the state is inferred by using presence records compiled in several databases.

In general, the studies on Mexican rattlesnakes have focused on their distribution at a large scale or on a single species. An example of this is the study by Paredes-García et al. (2011) about the representativeness of rattlesnakes in natural protected areas (NPAs), their natural history (Arnaud et al. 2008), ecology (Secor 2016), evolution (Strickland et al. 2018) and genetic diversity (Schield et al. 2018). Despite their importance in terms of ecology, public health and culture, it is generally considered that rattlesnakes have seen their populations significantly reduced, and the Mexican government confers different levels of protection for the nine species present in Zacatecas SEMARNAT (2019). According to the Red List published by IUCN (2020), the nine species are listed in the least concern (LC) risk category. Conservation threats mainly originate from the fragmentation of their habitat derived from land use changes, as well as hunting and illegal trade (Maritz et al. 2016), and indiscriminate killing due to the perceived danger associated with them (Ávila-Villegas 2017).

One way to determine distribution patterns is using species distribution modeling techniques (SDMs). These are based on the assumption that the distribution of a given species is the result -at least within a short time frame- of a balance between undisturbed factors, i.e., a (pseudo)-equilibrium between the biotic entities and the physical characteristics (Guisan and Theurillat 2000). Therefore, the SDMs are based on the environmental conditions of the sites where species are present (Phillips et al. 2006), representing the mathematical estimation of the ecological niche of the species in question and trying to establish the relationship between species distribution and the spatial distribution of the environmental variables utilized to generate the model (Elith et al. 2006). Distribution models are commonly used in several areas of biology, including biodiversity evaluations at various levels, so as to prioritize species conservation, in addition to the fields of evolutionary biology, epidemiology and global change biology (Araújo and Peterson 2012).

Due to the lack of information on rattlesnakes of Zacatecas, their local importance and the concern by state agencies, we launched a study on the distribution patterns of the nine species of rattlesnakes in Zacatecas to be used in rattlesnake management and conservation in the state. The aim of this paper is to combine the literature, field work and Species Distribution Models or SDMs to effectively determine the known and potential presence of these species in Zacatecas, to estimate *Crotalus* diversity per municipality, to identify their environmental requirements and gain knowledge about their biology.

## Methods

### Area of study

**Figure 2.**
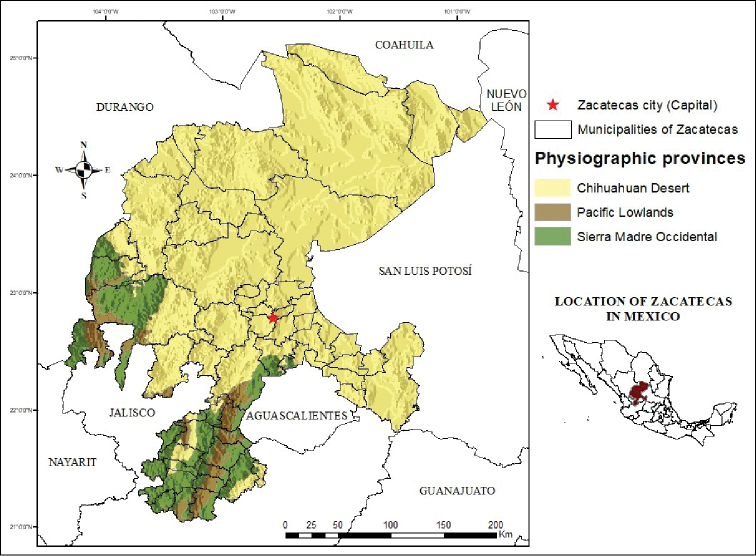
Location of Zacatecas in central-northern Mexico and Physiographic Provinces in which this state is situated (map based on Morrone et al. 2017).

The state of Zacatecas is located in Mexico’s central northern region (Figure 1), representing 3.7 % of the country’s surface area. The altitude in the state ranges from 800 to 3,120 meters above sea level (INEGI 2017). The climate in the central region of the state is cold and semi-arid, whereas the climate in the northwestern region is hot and also semi-arid (INEGI 2008). Conversely, the climate in the northeastern portion of Zacatecas is hot and arid, with a mean annual temperature of 19 °C and an annual rainfall of 289 mm. Finally, the area filled with ravines towards the southwestern region of the state has a warm, Mediterranean-like climate during the summer. This is the bioclimatic region that most highly contrasts with the rest of the state (Climate Data 2018). This climate diversity results in four biogeographical provinces that meet within the state (Figure 2): Chihuahuan Desert province, Pacific Lowlands province and Sierra Madre Occidental province (Morrone et al. 2017).

### Gathering presence records

To determine the potential distribution of rattlesnakes in Zacatecas, we gathered records on their global presence. In order to do this, we conducted queries in the databases of the Global Biodiversity Information Facility: GBIF 2016; Vertebrate Networks: Vertnet 2016 and the National Commission for the Knowledge and Use of Biodiversity: CONABIO 2016. Specialized literature also provided presence records of *Crotalus aquilus* (Carbajal-Márquez et al. 2015a) and *C. basiliscus* in Zacatecas (Ahumada-Carrillo et al. 2011; Carbajal-Márquez et al. 2015b). Finally, we incorporated the presence records reported by Ávila-Herrera (2012), Ávila-Herrera et al. (2020), Esparza-Estrada (2014)Lara-Galván (2015) and our own field work. The total number of records was 12,113, which were subsequently reduced to 3,412 (Table 1) by eliminating duplicated, inaccurate or incomplete records. We also considered records containing complementary information, such as the location, species biology and information strictly referring to the site where organisms were observed in order to use that in the geo-referencing process. Of the total number of presence records used in the potential distribution models, 248 correspond to the state of Zacatecas.

**Table 1.** Number of presence records shown by species of the *Crotalus* genus in Zacatecas included in the potential distribution modeling.

Group Studied	Species	Total occurrence records	Records pertaining to Zacatecas
Endemic to Mexico	*C. aquilus*	127	7
	*C. basiliscus*	115	3
	*C. polystictus*	90	14
Non-endemic to Mexico	*C. atrox*	999	39
	*C. lepidus*	375	52
	*C. molossus*	494	67
	*C. pricei*	117	2
	*C. scutulatus*	1,011	61
	*C. willardi*	84	3

### Definition of the spatial extent for modeling

According to Elith et al. (2011), when the study area or landscape of interest (L) is determined, it must contain a geographical area suggested and defined by the ecologist in relation to the problem, which can be delimited by geographical borders or by the knowledge of the point up to where the focal species may have spread. In addition, Merow et al. (2013) mentioned the importance of obtaining presence records for the species to be modeled, covering all the possible habitats where they might be found and the known distribution range of the species in question. Hence, the modelled presence records comprised the target species’ global distributions, and two different polygons that delimited the modelling extent. Thus, based on the IUCN (2020) classification for the species in the *Crotalus* genus reported in Zacatecas, two groups of study were established: 1) species endemic to Mexico and 2) species non-endemic to Mexico (Table 1). Based on this, the first polygon included the entire Mexican territory and was used to model the distribution of species endemic to Mexico (*Crotalus aquilus*, *C. basiliscus* and *C. polystictus*). The second polygon comprised the entire Mexican territory together with the southernmost states of the United States of America, namely, Arizona, Arkansas, California, Colorado, Kansas, Louisiana, Nevada, New Mexico, Oklahoma, Texas and Utah. This was done to include those species whose distribution range is also present in this portion of the USA (*Crotalus atrox*, *C. lepidus*, *C. molossus*, *C. pricei*, *C. scutulatus* and *C. willardi*).

### Species Distribution Modeling (SDM)

We prepared distribution models using package *sdm*, version 1.0.46, implemented in the R software, which assembles and evaluates models using 15 algorithms: BIOCLIM, BIOCLIM.DISMO, BRT, CART, FDA, GAM, GLM, GLMNET, MARS, MAXENT, MAXLIKE, MDF, RF, RPART and SVM. This provides the potential distribution of a given species or community. For each species, we used all available presence points, in addition to several background points created randomly within the modelling extent, so that the proportion of presence points was 30 % for all species (Naimi and Araújo 2016).

### Predictor variable selection

We utilized a set of environmental variables that are typically associated with the presence of the species, the 19 bioclimatic variables from the WorldClim, version 2.0 database (Fick and Hijmans 2017). These data refer to the minimum, median and maximum temperature, as well as to the rainfall records from 1970 to 2000 and any deviations associated with these data. These variables have a spatial resolution of 30’’ or approximately 1 km^2^. For the Mexican endemic species polygon, in addition to environmental variables, we included information on the land use, series V vegetation and edaphology (CONABIO 2017), rock types, topoform systems and the Mexican digital elevation continuum 3.0 (CEM 3.0). Further data was obtained from the National Statistics and Geography Institute (INEGI 2017), from which the slope and exposure were calculated. As for the non-endemic species polygon (Mexico and southern USA), we also included soil cover, human influence and the digital elevation continuum, downloaded from the Commission for Environmental Cooperation (CEC 2017), where the slope and exposure were also computed. Cartographic data was re-projected into the WGS_1984 world geographical coordinate system. The data of each predictor variable was cut out and retrieved. Map images were processed using ArcGIS 10.5 (ESRI 2016), QGIS 2.18.14 (QGIS 2009) and R 3.5.0 (R Core Team 2016).

Regarding variable selection (Table 2), we determined the most suitable variables for each species distribution model using the *multGLM* function in the *fuzzySim* R package (Barbosa 2015), where for each pair of correlated variables above |r| = 0.8, the function excludes the variable having the least significant relationship with the distribution of the species in question. The remaining variables are then subject to a stepwise selection process using the Akaike Information Criterion (AIC), to keep only informative variables in the models. Finally, any non-significant variables that might remain in the model after this process were eliminated (Reino et al. 2017; Gutiérrez-Rodríguez et al. 2017). The variables selected for each species were subsequently used to create models using the 15 algorithms applied in the *sdm* package. Afterwards, we calculated the mean and variance for each model’s predictions. The mean was used to show the potential distribution for each species of *Crotalus*, while the variance was used to estimate the agreement between predictions of different algorithms (Naimi and Araújo 2016). Finally, a potential distribution map was created based on the mean, cutting out the data to the surface of the region of interest, i.e. the state of Zacatecas.

**Table 2.** Variables selected by the *multGLM* function in the *fuzzySim* R package and used in distribution modeling per species of rattlesnake present in Zacatecas, Mexico.

Group Studied	Species	Variables used in SDM
Endemic	*C. aquilus*	bio_04, bio_09, bio_18, rock types and topoform systems.
	*C. basiliscus*	bio_07, bio_14, bio_15, bio_19, topoform systems and slope.
	*C. polystictus*	bio_04, bio_10, bio_15, bio_16, slope and topoform systems.
Non-Endemic	*C. atrox*	bio_01, bio_02, bio_03, bio_05, bio_08, bio_09, bio_12, bio_14, bio_15, bio_19, altitude, human influence and land cover.
	*C. lepidus*	bio_02, bio_03, bio_05, bio_08, bio_11, bio_14, bio_15, human influence, land cover and slope.
	*C. molossus*	bio_02, bio_08, bio_11, bio_12, bio_14, bio_15, bio_18, bio_19, altitude, human influence and land cover.
	*C. pricei*	bio_02, bio_08, bio_14, bio_19, altitude, human influence and land cover.
	*C. scutulatus*	bio_01, bio_05, bio_07, bio_09, bio_12, bio_14, bio_19, altitude, human influence, slope and land cover.
	*C. willardi*	bio_01, bio_08, bio_09, bio_12, bio_14, bio_18, bio_19, altitude and human influence.

**Abbreviation notes**: (bio_01) annual mean temperature, (bio_02) mean diurnal range, (bio_03) isothermality, (bio_04) temperature seasonality, (bio_05) max temperature of warmest month, (bio_06) min temperature of coldest month, (bio_07) temperature annual range, (bio_08) mean temperature of wettest quarter, (bio_09) mean temperature of driest quarter , (bio_10) mean temperature of warmest quarter, (bio_11) mean temperature of coldest quarter, (bio_12) annual precipitation, (bio_13) precipitation of wettest month, (bio_14) precipitation of driest month, (bio_15) precipitation seasonality, (bio_16) precipitation of wettest quarter, (bio_17) precipitation of driest quarter, (bio_18) precipitation of warmest quarter, and (bio_19) precipitation of coldest quarter.

### Model evaluation

Model evaluation was based on the criterion of area under the curve (AUC), which is considered a standard method to evaluate the discrimination capacity (i.e. differentiating the locations where presence and non-presence has been recorded) of predictive distribution models, avoiding the subjectivity of choosing a classification threshold. The value of AUC depends on the ratio of presence and the size of the area to be modeled, as pinpointed by (Lobo et al. 2008), but we used a constant prevalence value of 30 % for all species (Naimi and Araújo 2016).

### Species at the municipal level

**Figure 3.**
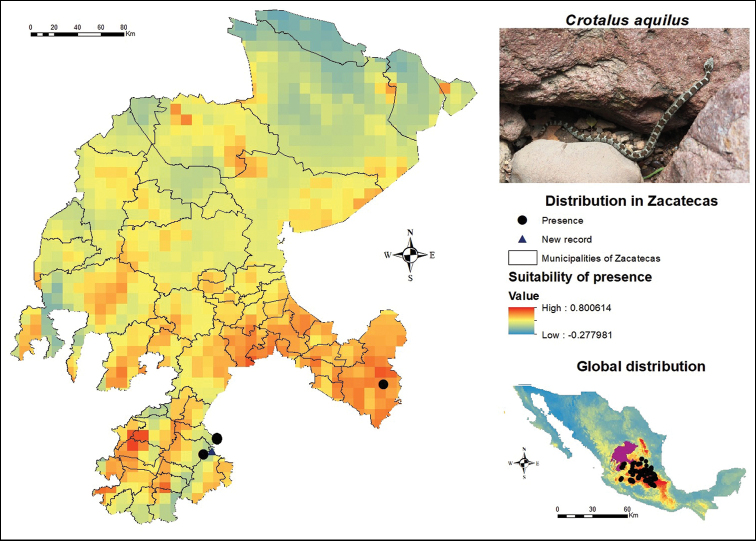
Presence and global potential distribution of *Crotalus aquilus* in Zacatecas, Mexico. Insert includes a *C. aquilus* from Tlachichila in the municipality of Nochistlán de Mejía (photo provided by Juan Felipe Martínez Montoya). The occurrence records used as input for the models are symbolized with dots, new records found during post-modeling field work are symbolized with triangles and the potential distribution is shown with warmer colors (red) identifying high potential for predicted presence for the species.

**Figure 4.**
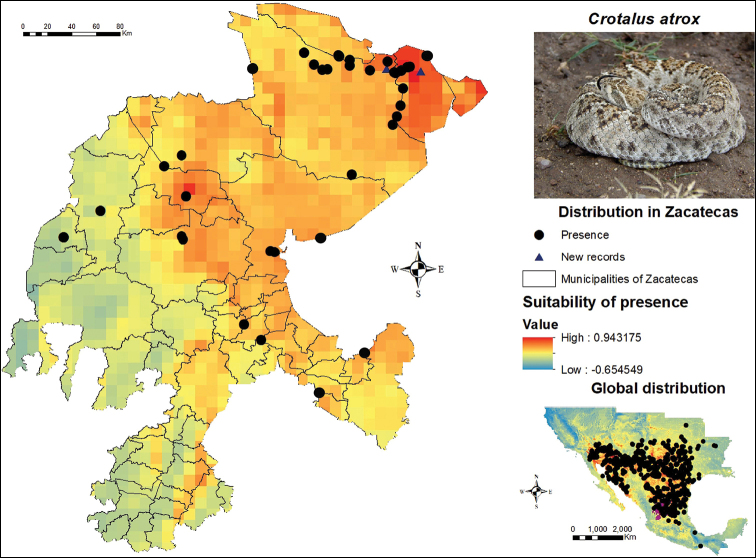
Presence and global potential distribution of *Crotalus atrox* in Zacatecas, Mexico. Insert includes a *C. atrox* from the municipality of Concepción del Oro (photo provided by Lenin Lara Galván). For explanation of the symbols and legend, see Figure 3.

**Figure 5.**
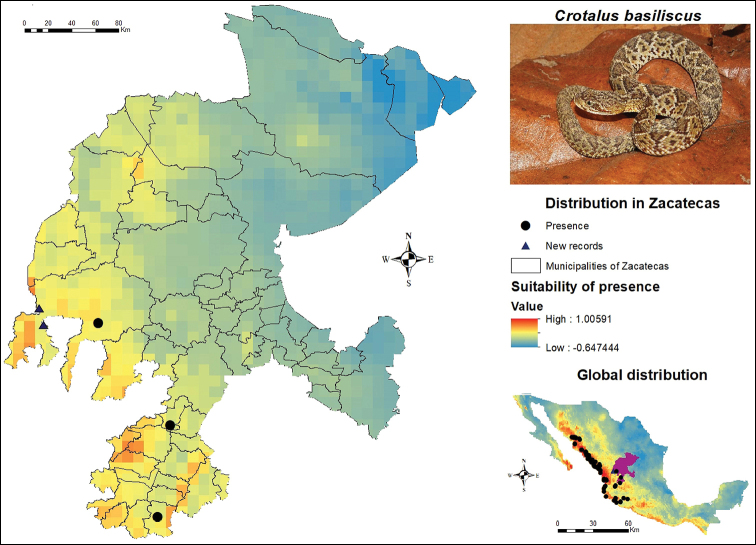
Presence and global potential distribution of *Crotalus basiliscus* in Zacatecas, Mexico. Insert includes a *C. basiliscus* from the municipality of Moyahua de Estrada (photo provided by Ivan Ahumada Carrillo). For explanation of the symbols and legend, see Figure 3.

**Figure 6.**
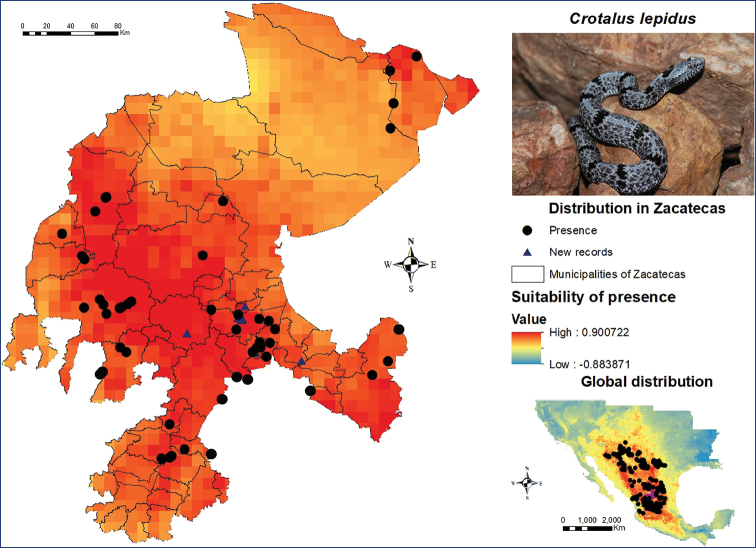
Presence and global potential distribution of *Crotalus lepidus* in Zacatecas, Mexico. Insert includes a *C. lepidus* from the semi-urban locality of Santa Monica in the municipality of Guadalupe (photo provided by Jesús Sigala Rodríguez). For explanation of the symbols and legend, see Figure 3.

**Figure 7.**
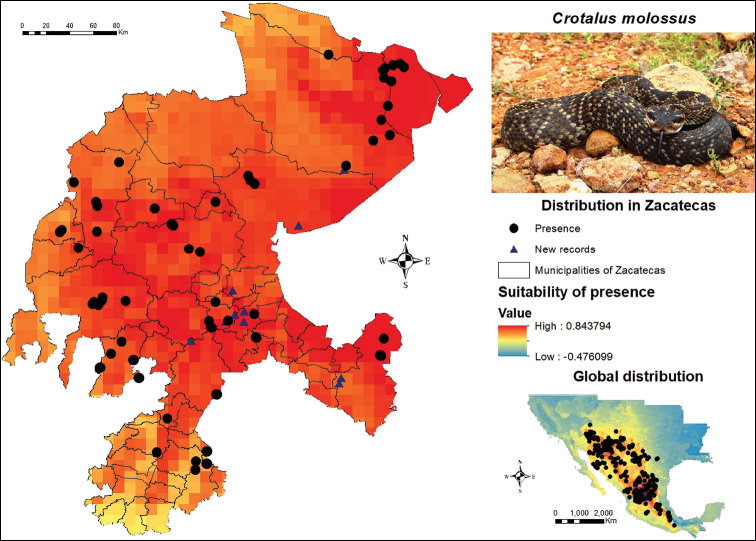
Presence and global potential distribution of *Crotalus molossus* in Zacatecas, Mexico. Insert includes a *C. molossus* from the municipality of Pinos (photo provided by Lenin Lara Galván). For explanation of the symbols and legend, see Figure 3.

**Figure 8.**
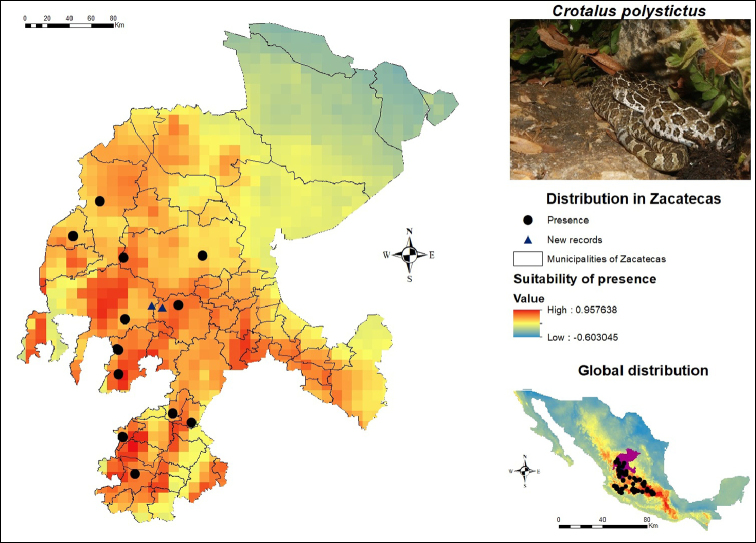
Presence and global potential distribution of *Crotalus polystictus* in Zacatecas, Mexico. Insert includes a *C. polystictus* from the municipality of Atolina (photo provided by Iván Ahumada Carrillo). For explanation of the symbols and legend, see Figure 3.

**Figure 9.**
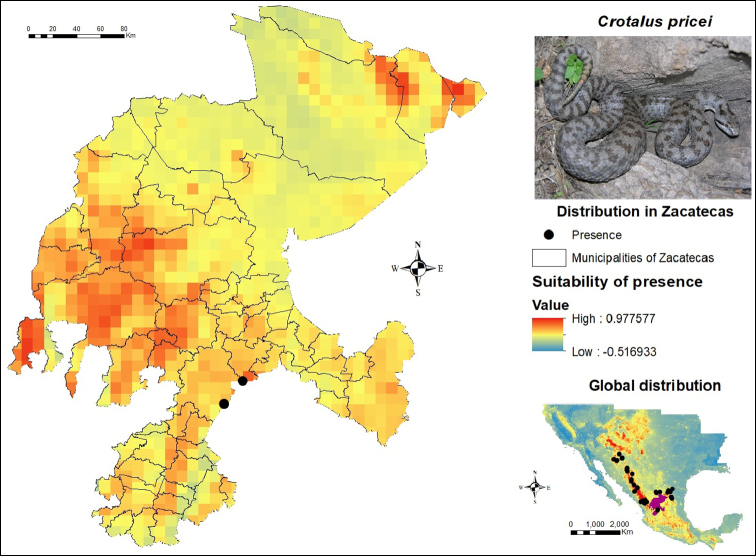
Presence and global potential distribution of *Crotalus pricei* in Zacatecas, Mexico. Insert includes a *C. pricei* from Durango state (photo provided by Jesús Sigala Rodríguez). For explanation of the symbols and legend, see Figure 3.

**Figure 10.**
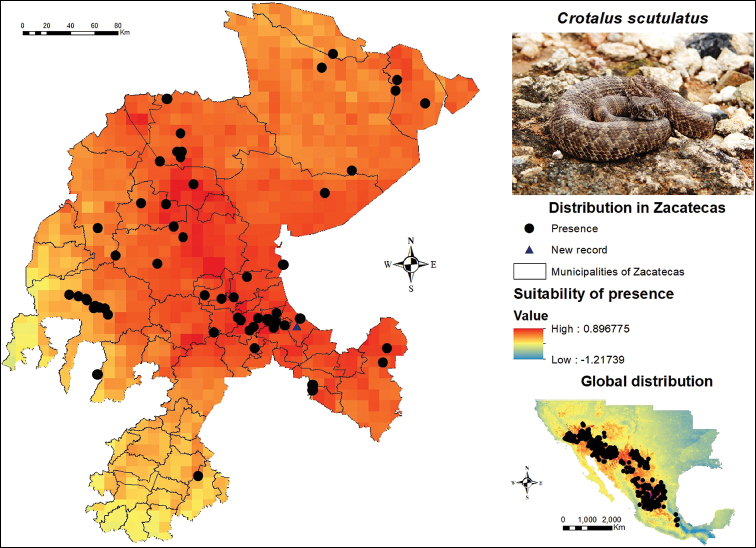
Presence and global potential distribution of *Crotalus scutulatus* in Zacatecas, Mexico. Insert includes a *C. scutulatus* from Pedregoso in the municipality of Pinos (photo provided by Lenin Lara Galván). For explanation of the symbols and legend, see Figure 3.

**Figure 11.**
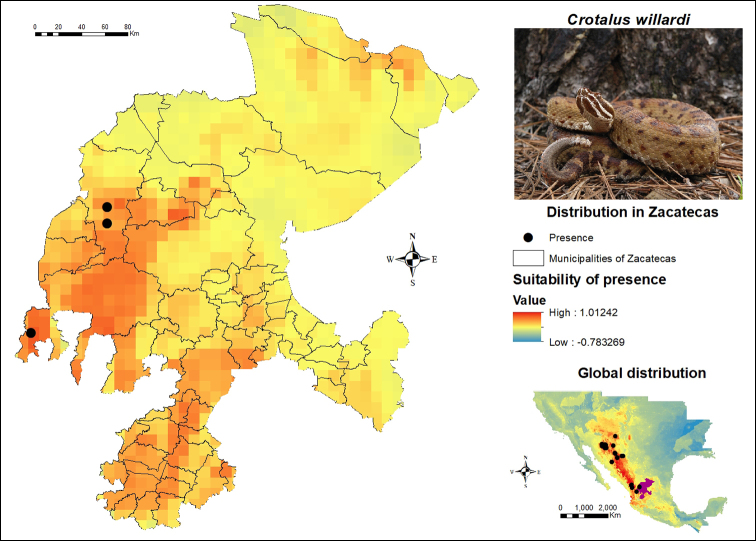
Presence and global potential distribution of *Crotalus willardi* in Zacatecas, Mexico. Insert includes a *C. willardi* from the state of Durango (photo provided by Joseph E. Forks). For explanation of the symbols and legend, see Figure 3.

To determine the rattlesnake number at the municipal level, we used the available presence records and prepared a list of the species observed at each municipality. Likewise, the list included the results of potential distribution models (Figures 3–11) that identified the areas bearing a higher suitability for these organisms. This implied overlapping the layer of municipalities over the potential distribution maps.

### Field verification of models

Based on the potential distribution models for each rattlesnake species found in Zacatecas, two localities per municipality were selected (three in the case of the Pinos, Loreto and Valparaíso municipalities). Thus, the field work in this study comprised a total of 48 localities throughout 22 municipalities within the state. These 22 municipalities were chosen randomly to include various regions throughout the state. Moreover, in these municipalities, we selected localities situated within the areas of greatest suitability as predicted by the models. A map of Mexican localities was overlapped to the potential distribution maps to conduct this geographical selection (CONABIO 2017). Field work was carried out in the areas surrounding the chosen localities in order to verify the presence of these species. To gather further information about these organisms, interviews were conducted to five individuals per locality, chosen randomly. Two of the five interviews targeted individuals who were believed to have closer contact with these organisms, i.e. individuals who engage in farming, cattle raising or rodent hunting. The other three interviews were carried out randomly, trying to include different age ranges and sexes. Finally, field verification was conducted in those areas having the most suitable habitats for the presence of these organisms based on the recommendations by Campbell and Lamar (2004), where the main criteria are related to land use, vegetation at elevation, including night driving along secondary roads, dirt roads and paths. At each location, we took into consideration visual records, skin sheds in good state, road kills and personal communications that could be confirmed (e.g. photographs and hunted specimens). A minimum sampling effort of 2 hours per location was considered, thus having a total of 243 hours of field work in a time period spanning from May to September of 2019, representing 22.09 field work hours/man.

## Results

There was significant variation on the number of records for the rattlesnakes present in Zacatecas, ranging from 1,011 records for *C. scutulatus* to 84 records for *C. willardi* throughout their distribution range. The latter species, along with *C. basiliscus* and *C. pricei*, had the lowest number of available presence records, as opposed to *C. molossus*, which was the species with the greatest number of records in Zacatecas (Table 1).

In the species distribution modeling, the most informative environmental variables for the models were bio_08 (mean temperature of wettest quarter), bio_14 (precipitation of driest month), bio_19 (precipitation of coldest quarter), altitude, land cover and slope. Anthropogenic influence, which intuitively includes the direct influence of human beings on ecosystems (based on population density, built-up area, highways, railroads, navigable rivers, land use and night-time illumination), impacted the presence of *C. atrox*, *C. lepidus*, *C. molossus and C. scutulatus*. Rock types and edaphology were less relevant at the time of model preparation. On the other hand, variables such as bio_06 (min temperature of coldest month) or bio_10 (mean temperature of warmest quarter) were not selected in any of the distribution models.

### Species Distribution Models -SDM-

In Zacatecas, the potential areas for the distribution of *C. aquilus* (Figure 3) are mainly found in the municipalities of Atolinga, Tepechitlán and Tlaltenango de Sánchez Román in the southwest. The distribution modeling also indicates areas in Genaro Codina, Cuauhtémoc and the municipality of Pinos, the latter in the southeastern region of the state. According to the distribution presented here, more organisms could be found in the border region between Aguascalientes and Zacatecas, since it presents a high degree of suitability for its occurrence, in addition to the municipalities of General Pánfilo Natera, Noria de Ángeles, Villa González Ortega and Villa Hidalgo that also present moderately high values. Regarding its global distribution, the largest number of records are located in the states of Guanajuato, Hidalgo and Querétaro, as well as specific records in Mexico City.

The western diamondback rattlesnake, *Crotalus atrox* is present in a large portion of the territory of Zacatecas (Figure 4), mainly along the northern region, which includes the municipalities of Concepción del Oro (where a record gathered during previous work is added), El Salvador and Mazapil. This species also showed records in the central and northern regions of the state, in the municipalities of Fresnillo and Río Grande, which exhibit distribution potential for the species. *C. atrox* has an extensive distribution in Zacatecas. During our post-modeling field work, many more individuals of this species were found in the municipalities of Concepción del Oro and Mazapil. The eastern region of Zacatecas has a low and medium suitability for this species, although a presence point is registered in the municipality of Chalchihuites. The southwestern region of Zacatecas stands out as the area having the lowest suitability throughout the entire state.

The known distribution of *C. basiliscus* in Zacatecas is limited to the western and southwestern parts of the state (Figure 5). This species has presence records in the municipalities of El Plateado de Joaquín Amaro, Moyahua de Estrada and Valparaíso. However, the SDM indicates the following municipalities as medium suitability areas: Apozol, Atolinga, Jalpa, Juchipila, Mezquital del Oro, Momax, Monte Escobedo, Nochistlán de Mejía, Tepechitlán, Tlaltenango de Sánchez Román and Trinidad García de la Cadena. Two presence records were added by post-modelling field work, including a live specimen caught and released, which was provided by Bañuelos-Alamillo (pers. comm. 2018), as well as the record of a dead specimen provided by a resident from the locality of San Rafael de las Tablas. Both records are located within the municipality of Valparaíso and the zones in question are included in the delimited areas with the greatest distribution potential.

The rock rattlesnake *Crotalus lepidus* is one of the species with most presence records in the state. The global potential distribution model for this species (Figure 6) contained a total of 375 occurrence records. Among these, 52 records are located in Zacatecas, mainly concentrating within the state’s central region, which is home to the largest urban areas and is composed of the municipalities of Guadalupe and Zacatecas. This species shows presence records primarily in the municipalities of Cuauhtémoc, Genaro Codina and Valparaíso. According to its global distribution model, its potential presence extends throughout the entire state of Zacatecas, from the municipalities of Pinos in the central region, where a significant number of records were identified, to the municipalities of Genaro Codina, Vetagrande and Villanueva. A few records were similarly identified in the northern region of the municipalities of Concepción del Oro, El Salvador, Cañitas de Felipe Pescador, General Francisco R. Murguía, Juan Aldama, Mazapil, Melchor Ocampo and Miguel Auza, in spite of showing a low suitability in the model. According to the SDM, the environment with the most suitable conditions for the presence of this species is located in the municipalities of Guadalupe, Jerez, Fresnillo, Monte Escobedo, Valparaíso, Vetagrande and Zacatecas. These rattlesnakes were found during post-modelling field work in the former two and the latter two municipalities. Two specimens were also spotted in the municipalities of Genaro Codina and Ojocaliente.

The black-tailed rattlesnake (*C. molossus*) exhibits an extensive potential distribution within Zacatecas (Figure 7). This species spreads throughout the whole state, from the municipality of Pinos in the southeastern region to Sombrerete in the western region, with a substantial number of records along the northern region, particularly in the municipalities of Concepción del Oro and El Salvador. Specimens of this species were discovered in the municipalities of Guadalupe, Mazapil, Pánuco, Villanueva, Villa de Cos and Zacatecas during field work. One more organism and a clearly identifiable record of a skin shed of this species were found in Pinos. When inhabitants from different localities in some municipalities of Zacatecas were interviewed, they mentioned the black-tailed rattlesnake as the most abundant and most commonly observed species in parcels, dirt roads and paved roads. This species, together with *C. atrox*, are usually sold for food in the state capital. Conversely, Mezquital del Oro, Moyahua de Estrada and Trinidad García were some of the municipalities presenting a medium suitability for *C. molossus*, although the possibility of finding them in these areas cannot be ruled out.

In the case of *C. polystictus*, the presence records (Figure 8) are concentrated in the western and southwestern region of the state, precisely in the municipalities Atolinga, Chalchihuites, El Plateado de Joaquín Amaro, Fresnillo, Jerez, Monte Escobedo, Sombrerete, Tabasco, Teúl de González Ortega and Valparaíso. Additional sources for *Crotalus polystictus* were consulted (Meik et al. 2012; Santiago-Pérez et al. 2017) to obtain a greater number of presence records. These zones also coincide with a high potential of suitability for their presence, according to the SDM. Besides, other regions of the municipalities of Apozol, Apulco, Juchipila, Tepechitlán and Genaro Codina were highlighted, the latter in the center-south of Zacatecas. On the other hand, the northern zone shows a low potential for its presence, so it is recommended to apply a greater sampling effort in the regions presented in red. In the central portion of the state, two new records for this species can be seen, which were provided by Bañuelos-Alamillo (2018). One is located in the municipality of Jerez and the other one in the nearby municipality of Valparaíso, both coinciding with an area of high suitability for *C. polystictus*.

Regarding its global distribution, it extends from Zacatecas to Mexico City and it is similarly reported in the states of Aguascalientes, Jalisco, Guanajuato, Michoacán, Querétaro and the State of Mexico, all of them exhibiting a significant number of records. In the southwestern portion of its known distribution, the record which is found closest to Colima was in the municipality of Tuxpan in Jalisco. Several authors mentioned that this species *probably occurs*Campbell and Lamar (2004) and is *likely to occur*Lemos-Espinal et al. (2020) in Colima. However, no presence records for this area were registered.

Unlike other species with a greater number of records, there were only two presence records of *Crotalus pricei* in Zacatecas, which are located in the municipalities of Genaro Codina and Villanueva. According to the SDM (Figure 9), the western region of Zacatecas presents the minimum essential ecological conditions for the presence of this species, mainly in the municipalities of Chalchihuites, the western portion of Fresnillo, Jerez, Jiménez del Teul, Monte Escobedo, the south of Sombrerete, Susticacán, the northern area of Tepetongo and especially the municipality of Valparaíso, which shows a high potential for its development. On the other hand, in the northern region of Zacatecas, these parameters were found in Concepción del Oro, El Salvador and Mazapil, which could be due to the occurrence records in the northeast region of its global distribution. This extends from Arizona in the southern United States, down to Mexico throughout the states of Sonora, Chihuahua and Durango, with the latter two being the states where the largest number of *C. pricei* records were concentrated, together with those located in the state of Nuevo León. In the center of Mexico, it is possible to find two specific records in San Luis Potosí, one mentioned by Grünwald et al. (2012) and an additional one included in the personal field work database of Jesús Sigala.

During the field work, no direct observations or skin sheddings were identified for this species, although there are two records a few kilometers from the Zacatecas border. One of them is located in the municipality of Mezquitic, Durango (Grünwald et al. 2012) and a more recent one is situated in Mezquital, also in the state of Durango, and included in the personal database of Jesús Sigala. In Zacatecas, this species can be considered rare, which evidences the necessity for increased field work efforts, especially at high altitudes, since the records hereby presented were found in areas > 2,500 m., thus lying within the altitude range reported by Campbell and Lamar (2004), precisely 1,850 – 3,203 m. Therefore, it is recommended to sample high elevation areas that coincide with the greatest potential for the distribution of this species in order to confirm its presence, as well as to increase the number of records and carry out a modeling based on the presence of this organism.

*Crotalus scutulatus* is one of the species with the greatest number of records for the construction of the SDM (Figure 10). The points of occurrence gathered are mainly located in the municipalities of Guadalupe and Zacatecas, spreading their distribution range both northwards into the municipalities of General Francisco R. Murguía, Fresnillo, Río Grande and Sain Alto, and southwards, starting from Ojocaliente and finishing in Pinos. Valparaíso concentrates a substantial amount of records for this species, which is also found on the other side of the state, namely, in the municipality of Concepción del Oro. During field work, this species was identified in the municipality of Pánfilo Natera in an area of abundant plateaus and barren soil. *C. scutulatus* is essentially present throughout the entire state, although the southern and southwestern regions have a medium potential suitability.

Finally, the occurrence data and the potential distribution map of *C. willardi* are shown in Figure 11. To prepare the modelling of this species throughout its known distribution range, 84 geo-referenced points were available. Among these, two were registered in Sombrerete and one in Valparaíso and according to the SDM, they are also the municipalities of Zacatecas that exhibit the greatest potential for their distribution. In addition, specific areas with high potential for its presence are shown in Fresnillo, Jiménez del Teul, Monte Escobedo and a series of municipalities in the southwestern region of the state. Along with *C. pricei*, this is one of the species with the lowest number of occurrence records in Zacatecas. Regarding their global distribution, the presence records are spread in the western region of Mexico, mainly in the states of Durango, Chihuahua and Sonora, as well as in Arizona and New Mexico in the United States. It is recommended to apply greater sampling efforts in Valparaíso and Sombrerete, especially in the Durango-Zacatecas border area in order to increase the number of localities for this species in the region.

### Species at the municipal level

Thirty-nine (67.24 %) out of the fifty-eight municipalities (Table 3) have at least one record of rattlesnake species. During the field verification performed in the potential distribution areas for these organisms in Zacatecas, twenty-seven new occurrence records belonging to six species (*Crotalus atrox*, *C. basiliscus*, *C. lepidus*, *C. molossus*, *C. polysticus* and *C. scutulatus*) and one more unpublished record for *C. aquilus* were added. According to the compilation of total records, Sombrerete and Valparaíso were the municipalities exhibiting the highest rattlesnake diversity, with six different species being reported, followed by Fresnillo, with five different species. The municipalities of Chalchihuites, Concepción del Oro, El Plateado de Joaquín Amaro, Genaro Codina, Guadalupe, Mazapil, Monte Escobedo and Pinos reported four species each. The municipality of Zacatecas, where the state capital is located, has the presence of three species of this genus. Although there are no occurrence records for rattlesnakes in 19 municipalities, according to the SDM, all municipalities have the minimum conditions for the presence of rattlesnakes. It is noteworthy that *C. molossus* has reported presence in 24 municipalities and *C. lepidus* and *C. scutulatus* in 23 municipalities, as opposed to *C. pricei* and *C. willardi*, reported in only two municipalities.

**Table 3.** Rattlesnake diversity per municipality in Zacatecas, Mexico, based on published records, collection records and field work *Ca*: *Crotalus aquilus*, *Cax*: *C. atrox*, *Cb*: *C. basiliscus*, *Cl*: *C. lepidus*, *Cm*: *C. molossus*, *Cp*: *C. polystictus*, *Cpr*: *C. pricei*, *Cs*: *C. scutulatus*, and *Cw*: *C. willardi*.

Municipality	*Ca*	*Cax*	*Cb*	*Cl*	*Cm*	*Cp*	*Cpr*	*Cs*	*Cw*	Species
Apozol										–
Apulco										–
Atolinga						x				1
Benito Juárez										–
Calera de Víctor Rosales								x		1
Cañitas de Felipe Pescador				x	x					2
Concepción del Oro		x		x	x			x		4
Cuauhtémoc				x						1
Chalchihuites		x		x	x	x				4
El Plateado de Joaquín Amaro			x	x	x	x				4
El Salvador										–
Fresnillo		x		x	x	x		x		5
Genaro Codina				x	x		x	x		4
General Enrique Estrada										–
General Francisco R. Murguía		x						x		2
General Pánfilo Natera								x		1
Guadalupe		x		x	x			x		4
Huanusco	x			x	x					3
Jalpa				x	x					2
Jerez				x		x				2
Jiménez del Téul				x	x					2
Juan Aldama										–
Juchipila										–
Loreto		x								1
Luis Moya				x						1
Mazapil		x		x	x			x		4
Melchor Ocampo		x			x			x		3
Mezquital del Oro										–
Miguel Auza										–
Momax										–
Monte Escobedo				x	x	x		x		4
Morelos					x			x		2
Moyahua de Estrada			x							1
Nochistlán de Mejía	x				x			x		3
Noria de Ángeles										–
Ojocaliente		x		x				x		3
Pánuco					x			x		2
Pinos	x			x	x			x		4
Río Grande		x						x		2
Santa María de la Paz										–
Sain Alto					x			x		2
Sombrerete		x		x	x	x		x	x	6
Susticacán										–
Tabasco						x				1
Tepechitlán										–
Tepetongo										–
Teúl de González Ortega						x				1
Tlaltenago de Sánchez Román				x	x					2
Trancoso				x				x		2
Trinidad García de la Cadena										–
Valparaíso			x	x	x	x		x	x	6
Vetagrande				x						1
Villa de Cos		x			x			x		3
Villa García										–
Villa González Ortega										–
Villa Hidalgo										–
Villanueva					x		x	x		3
Zacatecas				x	x			x		3
Total municipalities with presence per species:	3	12	3	23	24	10	2	23	2	

## Discussion

The model building area spanned the entire known distribution range of these species, so as to cover all possible habitats where the species might be found (Merow et al. 2013). However, the discussion on results focuses on the area of interest, i.e. the state of Zacatecas, as done by Elith et al. (2011), who refer to the study area as that one having the problem or interest for the ecologist. All this aimed at gathering a greater number of occurrence records for species as *C. aquilus*, *C. pricei* and *C. willardi*.

*C. aquilus* exhibited presence records in Huanusco, Nochistlán de Mejía and Pinos. Likewise, the SDM identified the municipalities of Cuauhtémoc and Genaro Codina as potential areas for the occurrence of this species and the municipality of Pinos was also indicated as a potential area of occurrence, which is demonstrated with the presence record reported for this area.

During our post-modelling field work, further occurrence records were collected. Moreover, when interviews were applied to individuals from different communities, *C. basiliscus* was pointed out as a frequently spotted species in the region. Given that this organism is easily mistaken for *C. molossus*, we only considered records backed up with specimens and the knowledge that *C. basiliscus* prefers zones around 1,000 meters above sea level, which are the sites where georeferencing data were obtained. This is in agreement with the information reported by Campbell and Lamar (2004), who stated that this species prefers ecotone zones between tropical deciduous forests and pine-oak forests. Our results suggest that the slope is one of the most important variables for the construction of the distribution model for this species.

Human influence was one of the most widely used variables for the construction of potential distribution models for *C. atrox*, *C. lepidus*, *C. molossus*, *C. scutulatus*, *C. pricei* and *C. willardi*, which is consistent with the literature and data obtained, since the first four species do not seem to have any problem to occupy habitats in close proximity to both urban and rural areas. In relation to this, presence records of *C. atrox* and *C. molossus* were obtained within populated areas, sightings of *C. lepidus* and *C. molossus* were also registered in the hills of Los Alamitos and La Virgen, which are located next to the largest and most populated urban area in the state. Sightings of *C. lepidus* in the aforementioned regions occurred in steep slopes and predominantly rocky soils, being the first variable (i.e. slope) among the ones selected for the construction of the SDM. Human presence is not an obstacle for the sighting of *C. atrox* and *C. scutulatus*, or at least for their transit in the vicinity of noisy urban areas with dense housing and transportation infrastructures. Indeed, some of the documented records for *C. atrox* are in paved and dirt roads. Moreover, Tay-Zavala et al. (2002) mentioned *C. atrox* and *C. scutulatus* as two of the most important species in medical terms in Mexico, due to the large amount of snakebites caused by them. Those two species presented the greatest number of presence records and their distribution range is extensive across the USA and Mexico, including the state of Zacatecas.

Records of *C. pricei* in Zacatecas are scarce and Campbell and Lamar (2004) did not mention the distribution of this species in Zacatecas. However, it is reported in the historical records of Klauber (1949) and in this same study, very nearby localities in the state of Durango are mentioned. In the neighboring state of Aguascalientes, this species is extremely rare according to Sigala-Rodríguez (2008). However, Bañuelos-Alamillo (pers, comm. 2018) mentioned a new record in 2017. The SDM points out regions in northeastern Zacatecas, specifically the municipalities of Concepción del Oro, El Salvador and Mazapil, as those with a high distribution potential. This may be due to the fact that these municipalities have mountain ranges whose altitudes are higher than 2,900 meters above sea level (INEGI 2017), with altitude being one of the variables used to construct the model of the species in question, which agrees with Campbell and Lamar (2004), who mentioned the preference of this species for high elevations. Another potential reason why these areas turned out to be potential for this species could be the occurrence records of *C. pricei* in northeastern Mexico.

*C. scutulatus* has a wide distribution throughout the state. However, there are areas in southwestern Zacatecas which, according to the SDM, have a medium potential for the development of this species. This may be due to the type of vegetation preferred by these organisms and, consequently, the type of climate, given that these regions are usually more humid, as they lie within the Juchipila and Tlaltenango canyons. This results in a topography that greatly differs from that in most of Zacatecas, which, according to Campbell and Lamar (2004), are the most suitable habitats for the settling of this species. In the case of the specimen found in the municipality of General Pánfilo Natera, the sampled area exhibited a rocky, eroded soil, where *Opuntia* spp. was the predominant crop.

No specimens were recorded for *C. willardi* during post-modelling field work. Yet, all the individuals who were interviewed to gather further location data, when being shown photographs of rattlesnakes from Zacatecas, mentioned having seen this species in the mountainous regions within the municipality of Valparaíso. The physical characteristics of this species, such as its color and the shape of its head, rattle and scales, is what allowed the locals to immediately discriminate it from the rest of species reported in the state. Although field sampling failed to provide additional presence records for this species, the habitat, environment and altitudinal range in those regions in Valparaiso are coincident with the characteristics that Campbell and Lamar (2004) mention for this species.

There are no occurrence records for any rattlesnake species in 19 of the municipalities in Zacatecas (Table 3). However, it is important to point out that this does not mean that no records might be found in these zones, since according to the outputs produced by the distribution models for these species, all the municipalities in the state have appropriate conditions for the presence of at least one rattlesnake species. Zacatecas is one of the most extensive states in the central-northern part of Mexico; hence, additional sampling efforts are required to report new records within the state and SDM predictions can be a valuable tool to guide sampling effort in an efficient and cost-effective manner.

## Conclusions

Based on the obtained records and the field work, the distribution of nine rattlesnake species (*Crotalus* genus) was confirmed in the state of Zacatecas: *C. aquilus*, *C. atrox*, *C. basiliscus*, *C. lepidus*, *C. molossus*, *C. polystictus*, *C. pricei*, *C. scutulatus* and *C. willardi*. In addition to the confirmed records, there is available indirect information on their presence in many areas, including personal communications, unverified sightings and detailed descriptions of specimens matching the physical characteristics and ecological requirements of the species. However, it is necessary to confirm their presence in these zones.

We recommend applying greater field work efforts in areas where no occurrence records have yet been identified for these species, as well as for organisms that showed the lowest number of records, particularly in the areas with a higher potential for presence. Likewise, we strongly advise conducting field work during the July-October period, since this will increase the sighting probability. We also suggest updating models once additional occurrence records have been registered.

Results obtained from the distribution modeling of these species generally agree with field work verification, making it possible to prioritize field efforts more effectively in different localities. During field work verification, multiple night sightings of rattlesnakes were noted, both alive or run over by vehicles on the roads, being the latter one of the major causes of death of these species in the state. This piece of information was merely gathered for *Crotalus molossus* and *C. atrox*, species, which were found in these areas.

This paper represents substantial contribution to the knowledge on rattlesnakes in Zacatecas. Their occurrence records are shown and zones with greatest diversity within the state are inferred. Moreover, this study indicates the potential distribution areas of these organisms. This material will be of major help for the implementation of strategies on public health issues, as well as for the proposal of management and conservation plans for these species.

## Suggestions

We believe that this study could be used by individuals wishing to conduct future projects related to rattlesnake conservation in Zacatecas. We also encourage the publication and incorporation of new occurrence records that contribute to gather further information on these organisms in the state.

## Supplementary materials

Supplementary material 1 SF1 *Crotalus aquilus* global distribution Data type Image Explanation note Global model of potential distribution. https://binary.pensoft.net/file/488621This dataset is made available under the Open Database License (http://opendatacommons.org/licenses/odbl/1.0/). The Open Database License (ODbL) is a license agreement intended to allow users to freely share, modify, and use this Dataset while maintaining this same freedom for others, provided that the original source and author(s) are credited.Jesús Lenin Lara-Galván, Juan Felipe Martínez-Montoya, A. Márcia Barbosa

Supplementary material 2 SF2 *Crotalus atrox* global distribution Data type Image Explanation note Global model of potential distribution. https://binary.pensoft.net/file/488622This dataset is made available under the Open Database License (http://opendatacommons.org/licenses/odbl/1.0/). The Open Database License (ODbL) is a license agreement intended to allow users to freely share, modify, and use this Dataset while maintaining this same freedom for others, provided that the original source and author(s) are credited.Jesús Lenin Lara-Galván, Juan Felipe Martínez-Montoya, A. Márcia Barbosa

Supplementary material 3 SF3 *Crotalus basiliscus* global distribution Data type Image Explanation note Global model of potential distribution. https://binary.pensoft.net/file/488623This dataset is made available under the Open Database License (http://opendatacommons.org/licenses/odbl/1.0/). The Open Database License (ODbL) is a license agreement intended to allow users to freely share, modify, and use this Dataset while maintaining this same freedom for others, provided that the original source and author(s) are credited.Jesús Lenin Lara-Galván, Juan Felipe Martínez-Montoya, A. Márcia Barbosa

Supplementary material 4 SF4 *Crotalus lepidus* global distribution Data type Image Explanation note Global model of potential distribution. https://binary.pensoft.net/file/488624This dataset is made available under the Open Database License (http://opendatacommons.org/licenses/odbl/1.0/). The Open Database License (ODbL) is a license agreement intended to allow users to freely share, modify, and use this Dataset while maintaining this same freedom for others, provided that the original source and author(s) are credited.Jesús Lenin Lara-Galván, Juan Felipe Martínez-Montoya, A. Márcia Barbosa

Supplementary material 5 SF5*Crotalus molossus* global distribution Data type Image Explanation note Global model of potential distribution. https://binary.pensoft.net/file/488625This dataset is made available under the Open Database License (http://opendatacommons.org/licenses/odbl/1.0/). The Open Database License (ODbL) is a license agreement intended to allow users to freely share, modify, and use this Dataset while maintaining this same freedom for others, provided that the original source and author(s) are credited.Jesús Lenin Lara-Galván, Juan Felipe Martínez-Montoya, A. Márcia Barbosa

Supplementary material 6 SF6 *Crotalus polystictus* global distribution Data type Image Explanation note Global model of potential distribution. https://binary.pensoft.net/file/488626This dataset is made available under the Open Database License (http://opendatacommons.org/licenses/odbl/1.0/). The Open Database License (ODbL) is a license agreement intended to allow users to freely share, modify, and use this Dataset while maintaining this same freedom for others, provided that the original source and author(s) are credited.Jesús Lenin Lara-Galván, Juan Felipe Martínez-Montoya, A. Márcia Barbosa

Supplementary material 7 SF7 *Crotalus pricei* global distribution Data type Image Explanation note Global model of potential distribution. https://binary.pensoft.net/file/488627This dataset is made available under the Open Database License (http://opendatacommons.org/licenses/odbl/1.0/). The Open Database License (ODbL) is a license agreement intended to allow users to freely share, modify, and use this Dataset while maintaining this same freedom for others, provided that the original source and author(s) are credited.Jesús Lenin Lara-Galván, Juan Felipe Martínez-Montoya, A. Márcia Barbosa

Supplementary material 8 SF8 *Crotalus scutulatus* global distribution Data type Image Explanation note Global model of potential distribution. https://binary.pensoft.net/file/488628This dataset is made available under the Open Database License (http://opendatacommons.org/licenses/odbl/1.0/). The Open Database License (ODbL) is a license agreement intended to allow users to freely share, modify, and use this Dataset while maintaining this same freedom for others, provided that the original source and author(s) are credited.Jesús Lenin Lara-Galván, Juan Felipe Martínez-Montoya, A. Márcia Barbosa

Supplementary material 9 SF9 *Crotalus willardi* global distribution Data type Image Explanation note Global model of potential distribution. https://binary.pensoft.net/file/488629This dataset is made available under the Open Database License (http://opendatacommons.org/licenses/odbl/1.0/). The Open Database License (ODbL) is a license agreement intended to allow users to freely share, modify, and use this Dataset while maintaining this same freedom for others, provided that the original source and author(s) are credited.Jesús Lenin Lara-Galván, Juan Felipe Martínez-Montoya, A. Márcia Barbosa
