# Supplementary figures and images for: Rattlesnake (Crotalus spp.) distribution and diversity in Zacatecas, Mexico
Source: Zookeys. 2020 Dec 18;1005:103–32. doi: 10.3897/zookeys.1005.56964 (PMC7762751; doi:10.3897/zookeys.1005.56964)

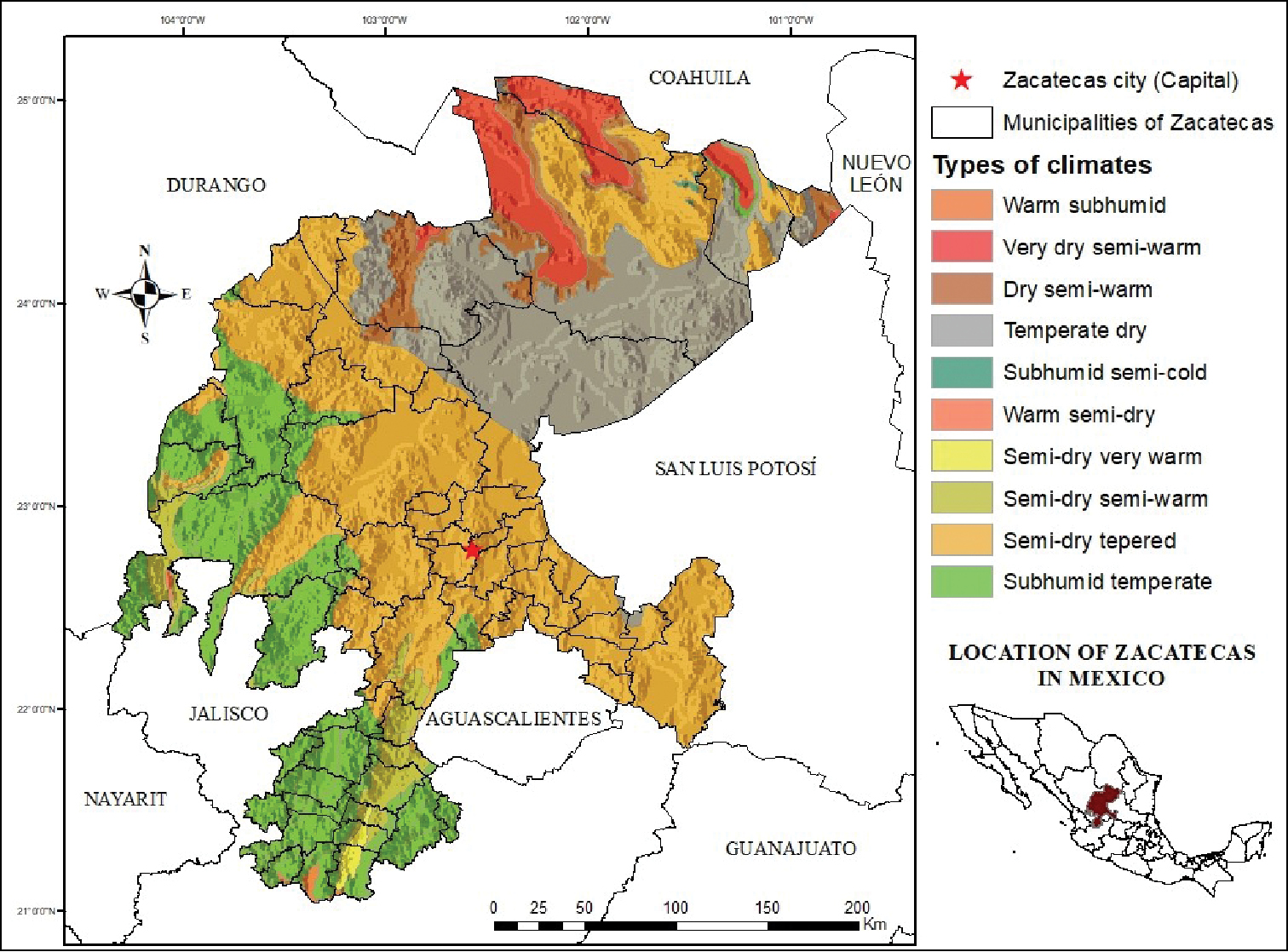

Supplement: Supplementary material 1 — SF1 Crotalus aquilus global distribution [file zookeys-1005-103-s001.jpg]

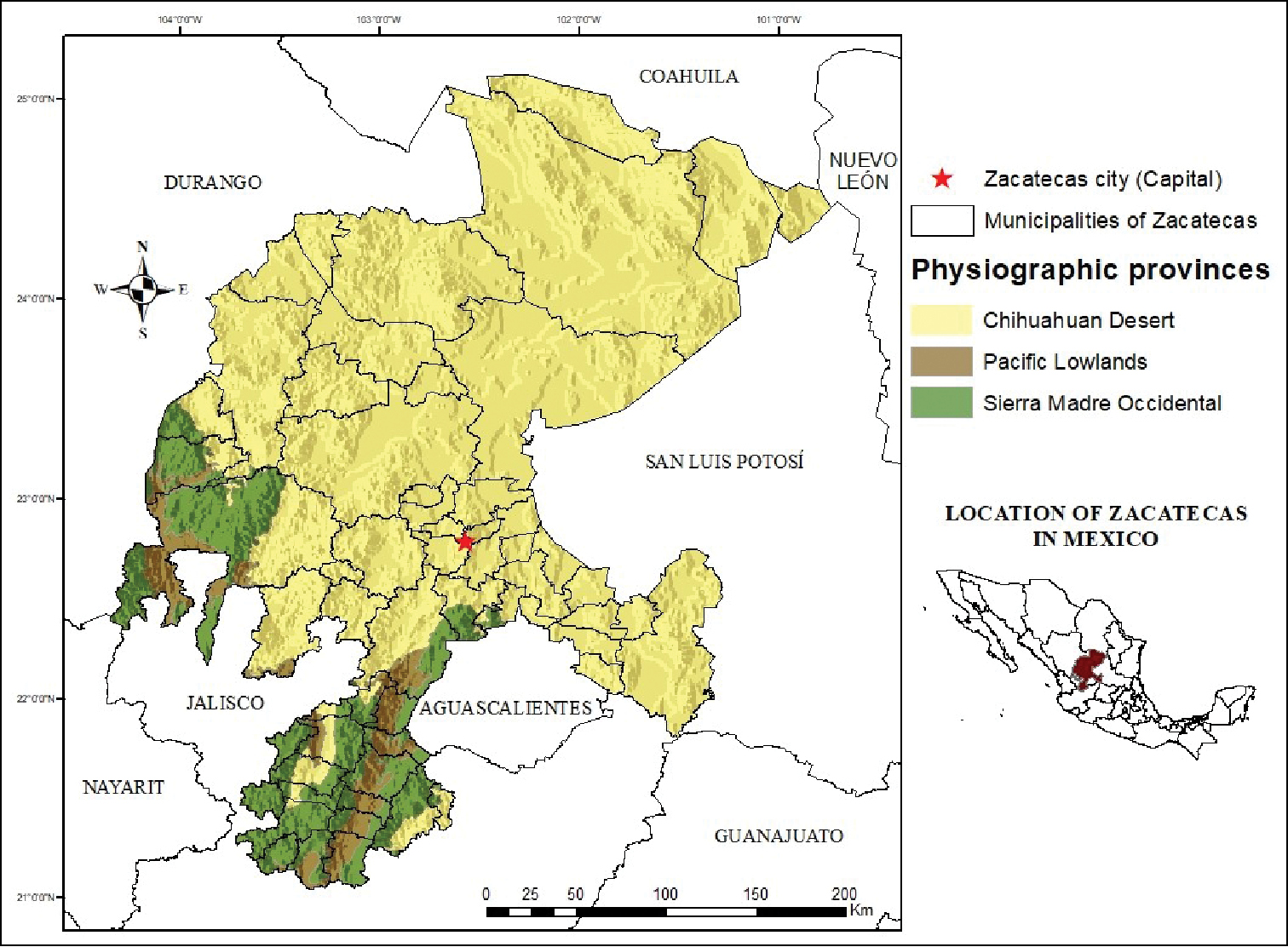

Supplement: Supplementary material 2 — SF2 Crotalus atrox global distribution [file zookeys-1005-103-s002.jpg]

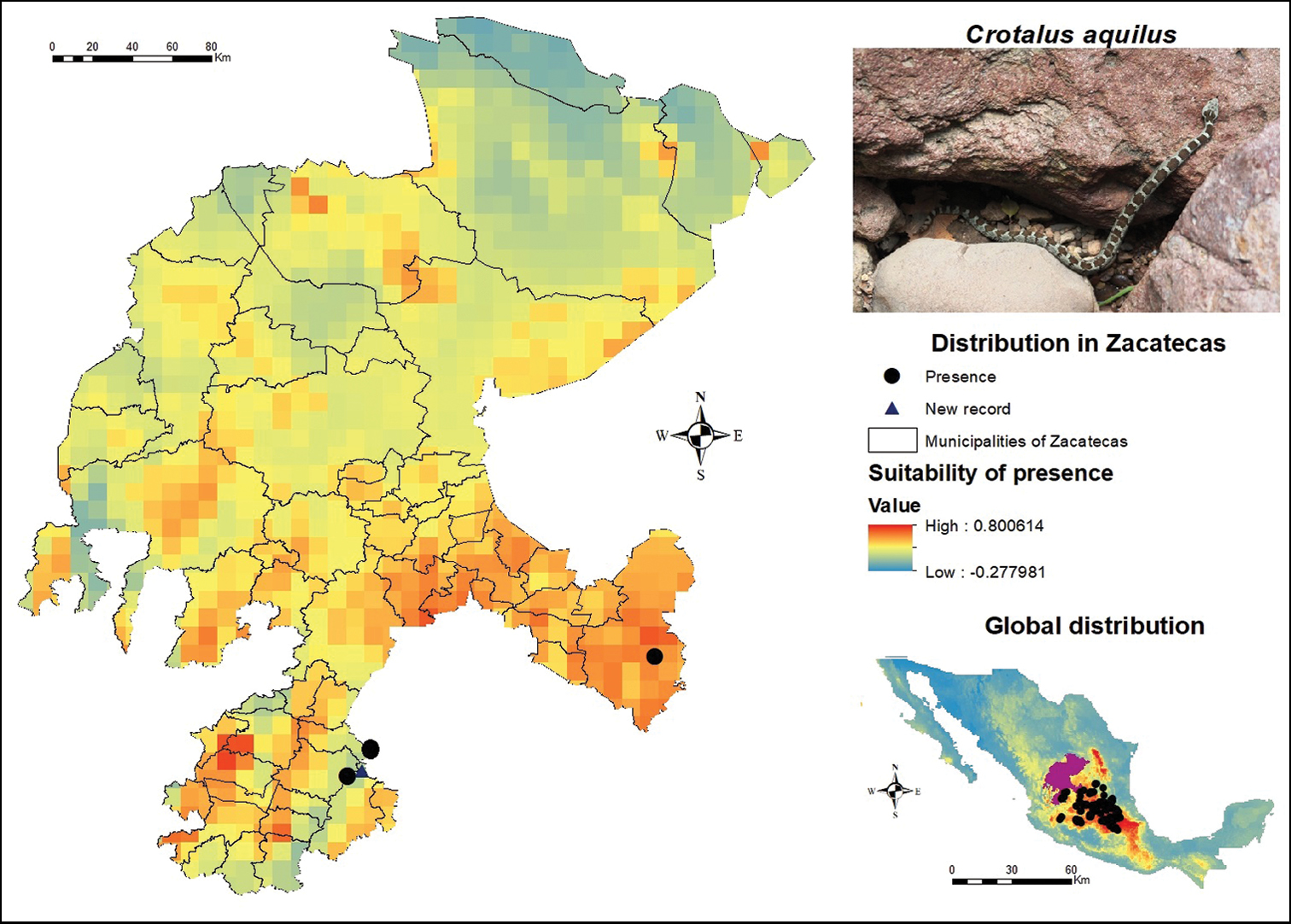

Supplement: Supplementary material 3 — SF3 Crotalus basiliscus global distribution [file zookeys-1005-103-s003.jpg]

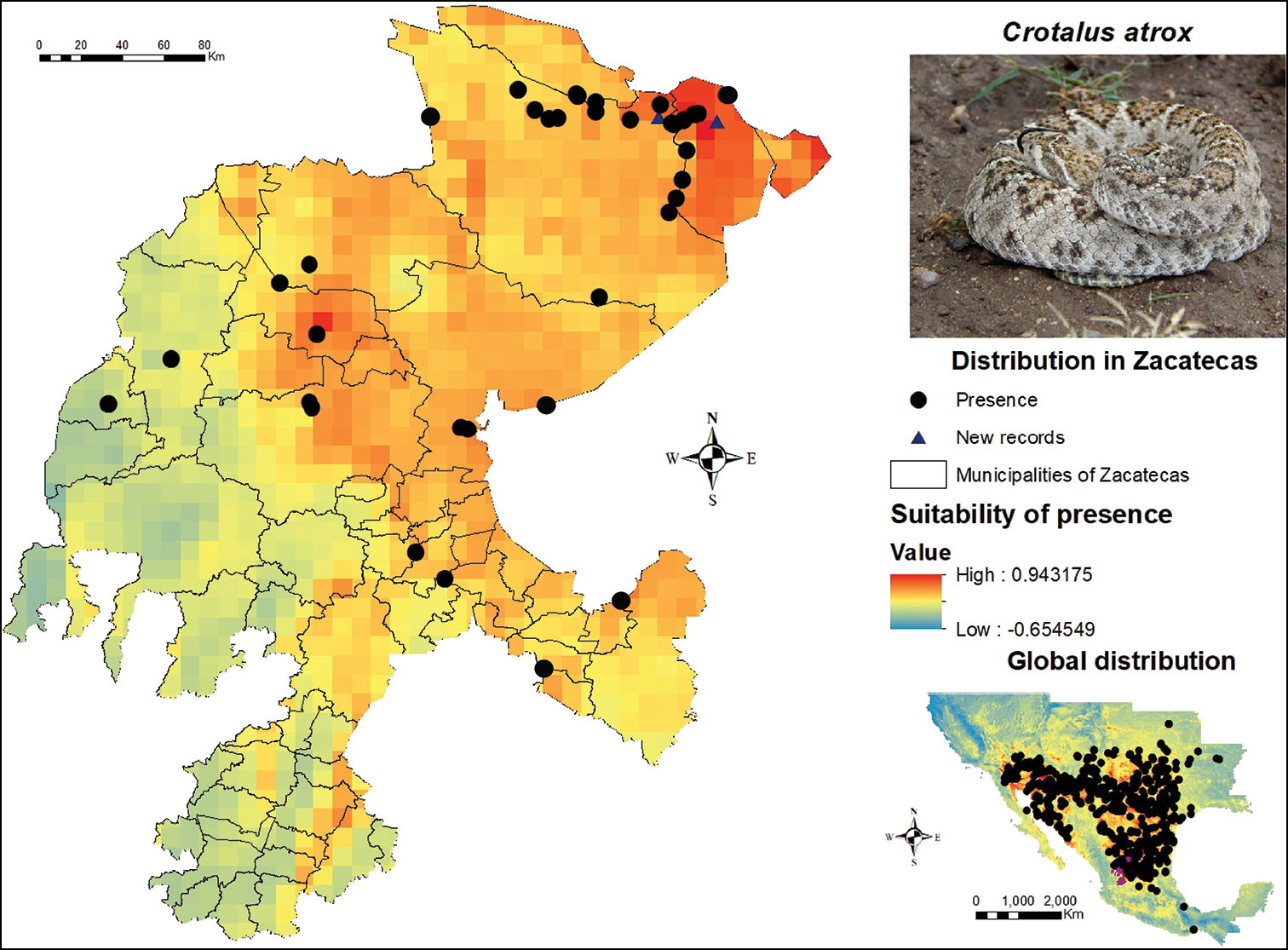

Supplement: Supplementary material 4 — SF4 Crotalus lepidus global distribution [file zookeys-1005-103-s004.jpg]

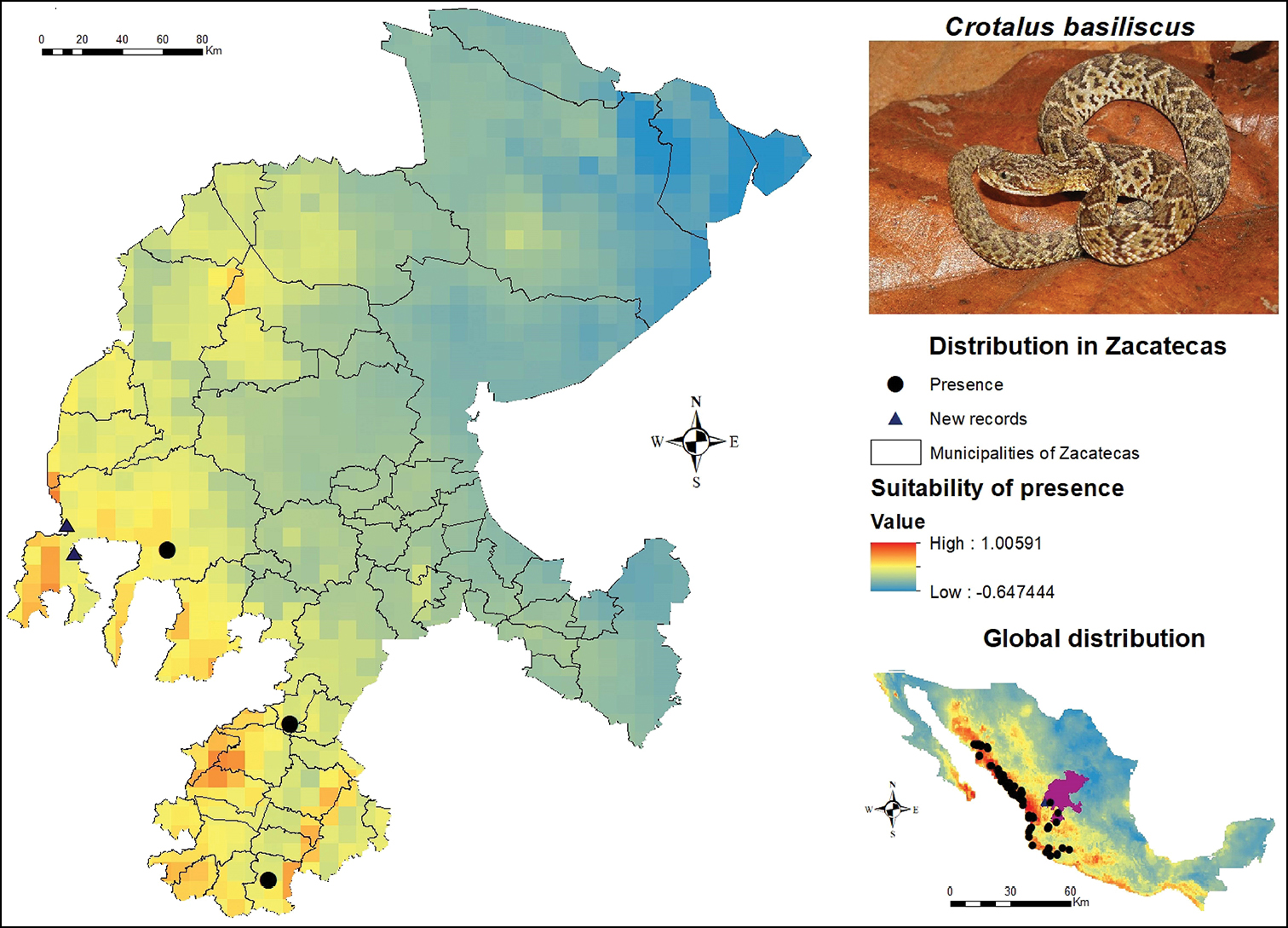

Supplement: Supplementary material 5 — SF5Crotalus molossus global distribution [file zookeys-1005-103-s005.jpg]

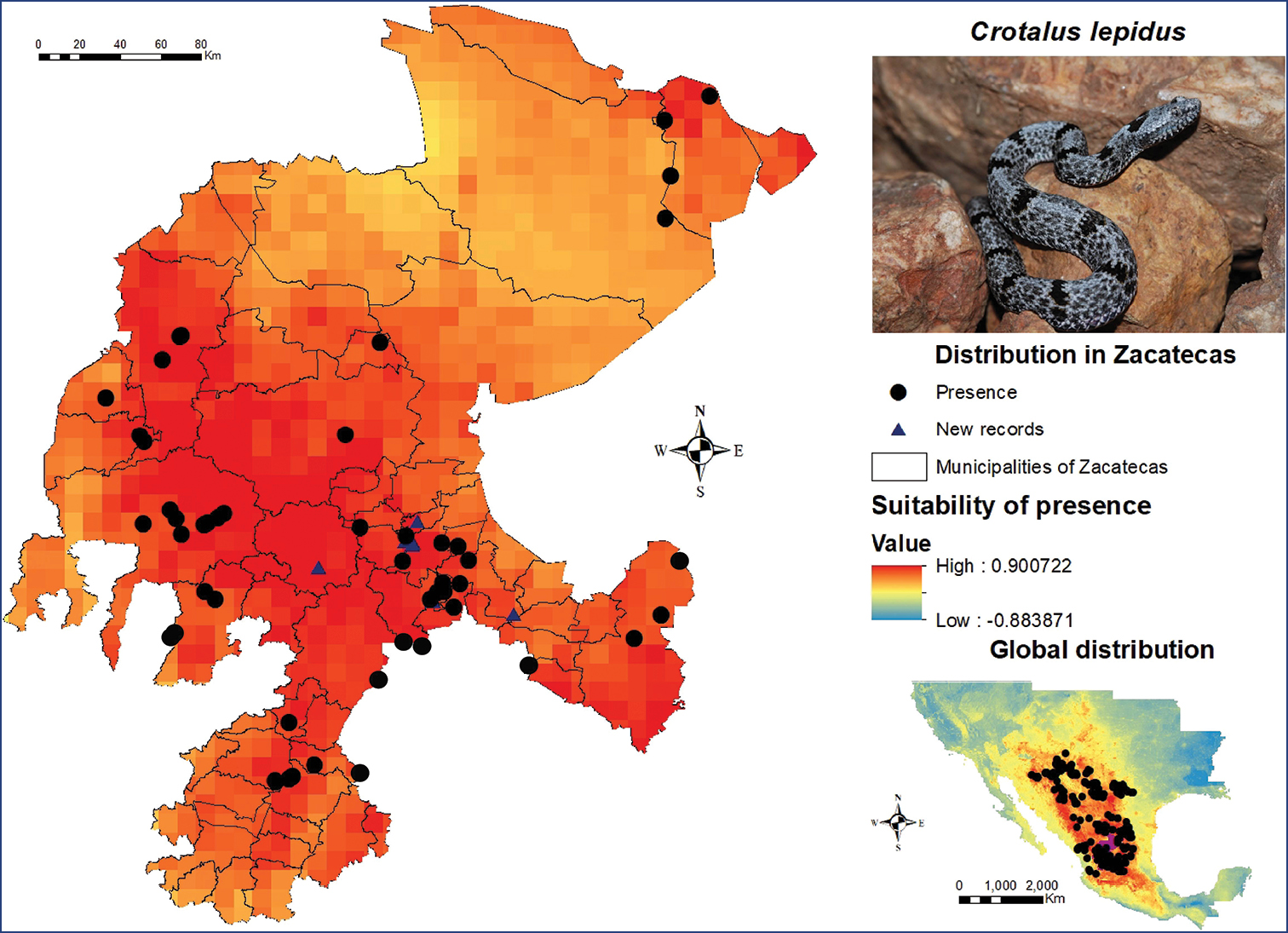

Supplement: Supplementary material 6 — SF6 Crotalus polystictus global distribution [file zookeys-1005-103-s006.jpg]

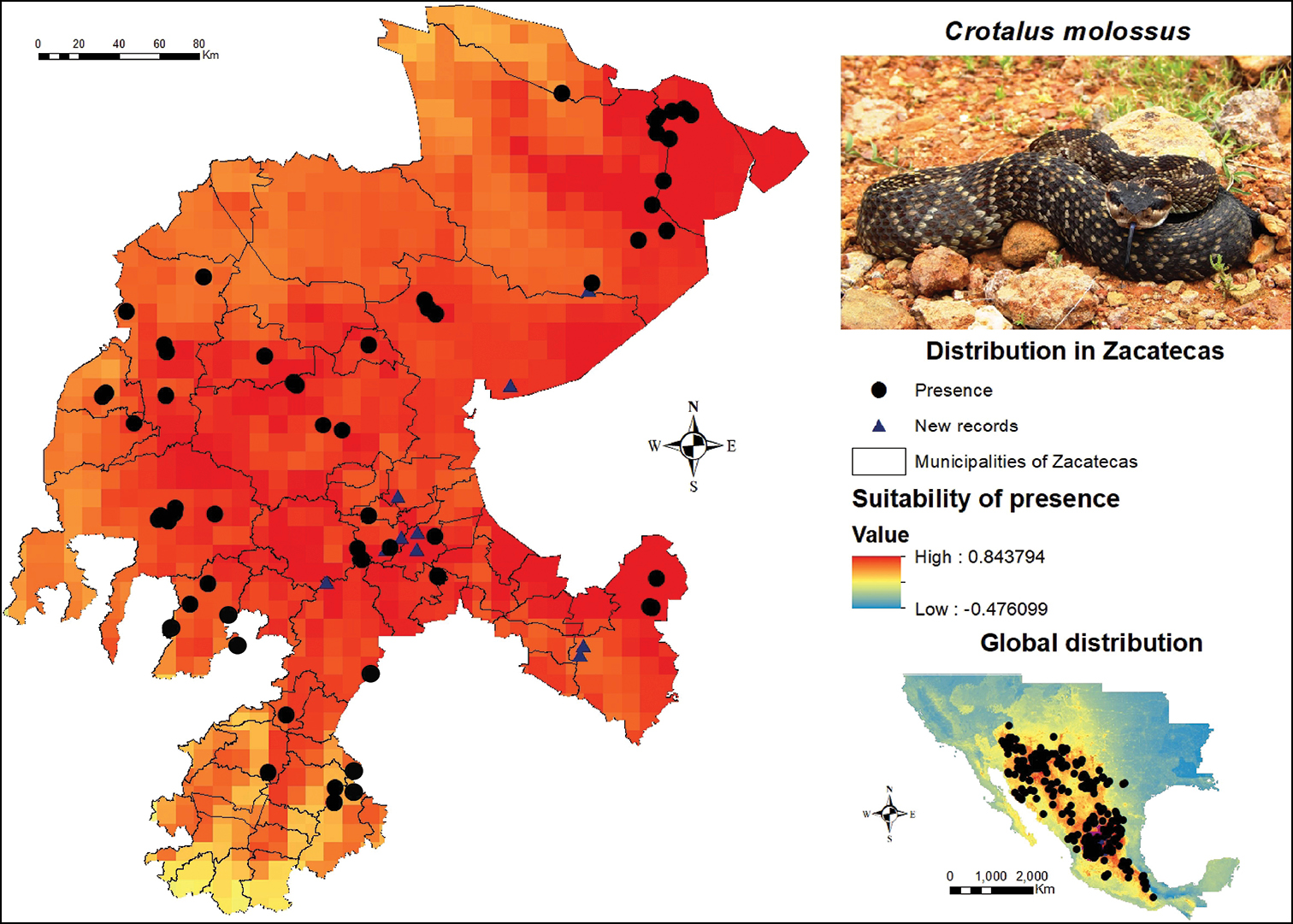

Supplement: Supplementary material 7 — SF7 Crotalus pricei global distribution [file zookeys-1005-103-s007.jpg]

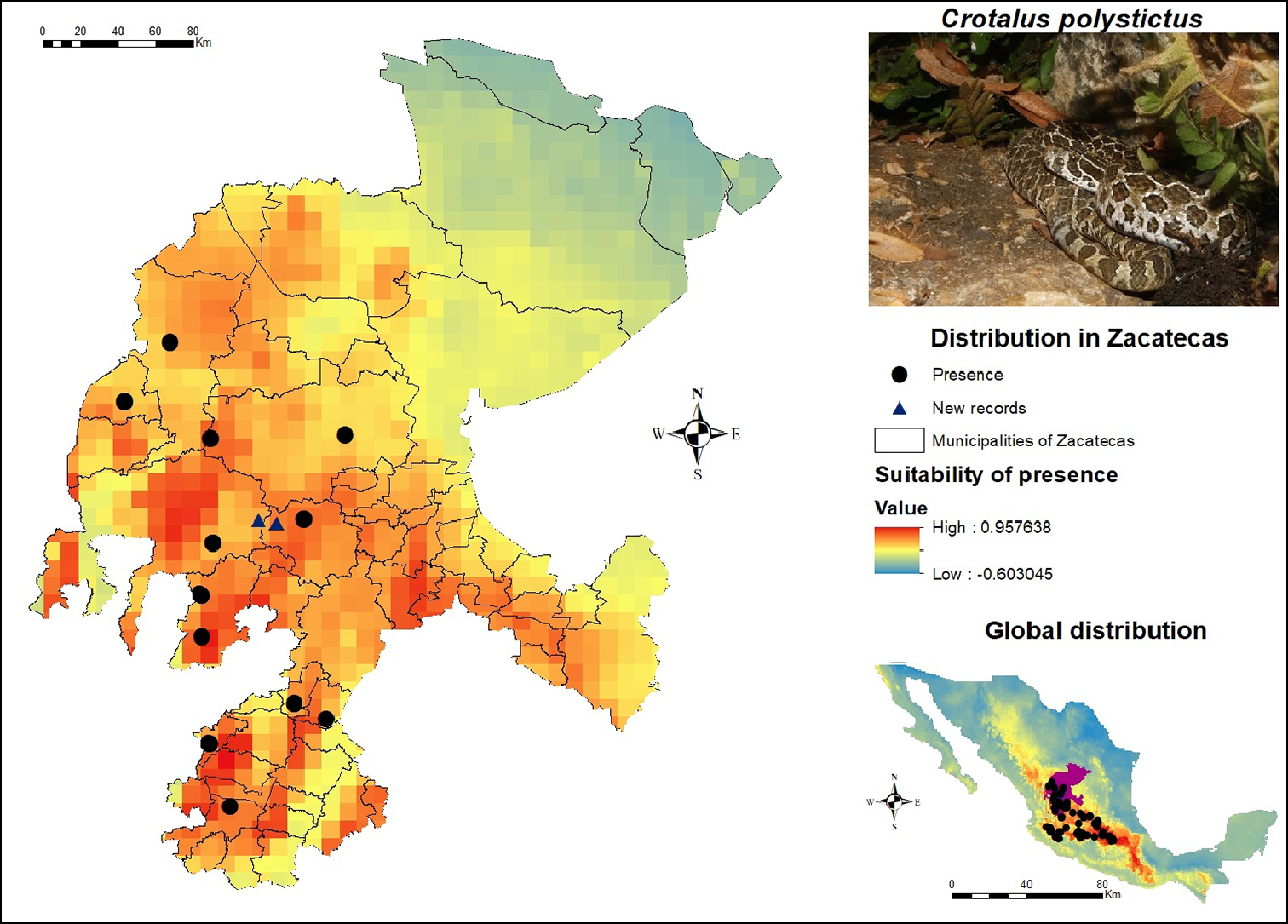

Supplement: Supplementary material 8 — SF8 Crotalus scutulatus global distribution [file zookeys-1005-103-s008.jpg]

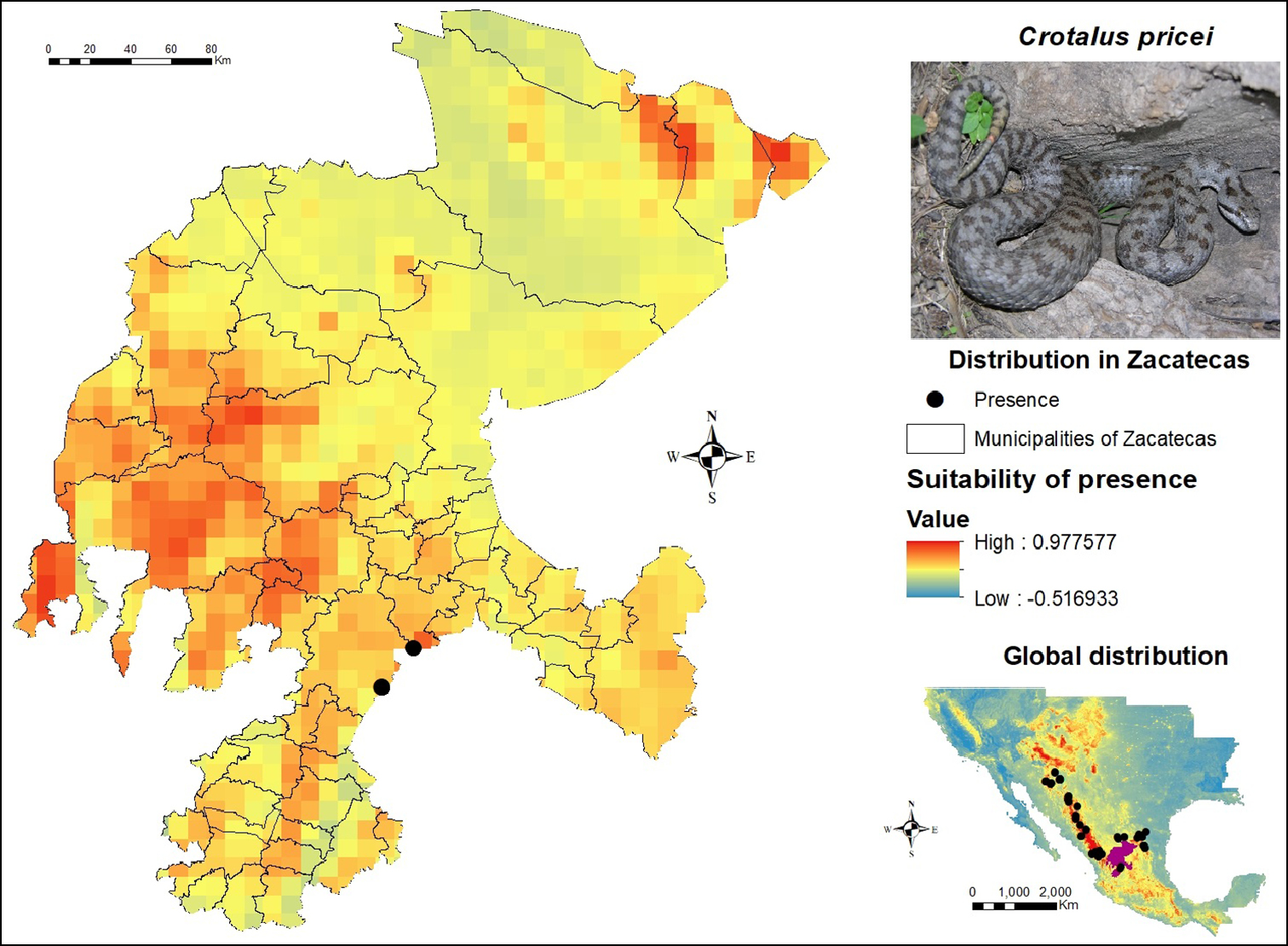

Supplement: Supplementary material 9 — SF9 Crotalus willardi global distribution [file zookeys-1005-103-s009.jpg]
